# Utility of the CapsoCam Plus for Overt Gastrointestinal Bleeding After Negative Gastroscopy: A Retrospective Cohort Study

**DOI:** 10.1155/grp/1813362

**Published:** 2026-11-09

**Authors:** Calvin M. Johnson, Veena Shivakumar, Stanley W. McKenzie, Reena Sidhu, Mark E. McAlindon

**Affiliations:** ^1^ Academic Unit of Gastroenterology and Hepatology Northern General Hospital Sheffield Teaching Hospitals Sheffield UK nhs.uk; ^2^ Division of Clinical Medicine School of Medicine and Population Health University of Sheffield Sheffield UK sheffield.ac.uk

**Keywords:** CapsoCam Plus, capsule endoscopy, endoscopy, gastrointestinal bleeding, small bowel

## Abstract

**Introduction:**

Patients with upper gastrointestinal bleeding have a nondiagnostic gastroscopy if bleeding is distal to the duodenum. Capsule endoscopy can localise bleeding to anywhere in the gastrointestinal tract; yield is highest when performed as soon as possible, although impaired motility in unwell patients reduces complete examination rate. The CapsoCam Plus operates without external support equipment, and has a long battery life, theoretically improving completion rate and diagnostic yield.

**Objectives:**

The objectives were to determine the examination completion rate (to the caecum), diagnostic yield and impact on management of the CapsoCam Plus in patients with suspected acute upper gastrointestinal bleeding and nondiagnostic gastroscopy.

**Methods:**

This was an observational retrospective cohort study of patients who underwent gastroscopy within 24 hours of admission to Sheffield Teaching Hospitals, with suspected upper gastrointestinal bleeding. Capsule endoscopy using the CapsoCam Plus was performed in those in whom gastroscopy was normal or identified lesions of uncertain significance. A single expert video reader identified and localised blood, pathological lesions, completeness of small bowel examination and duration of examination. Data were collected retrospectively, including the nature of bleeding, type and timing of investigations, management and diagnosis.

**Results:**

A median of 9 days (IQR 5–14 days) after bleeding, 113 capsule endoscopies were performed in 106 patients (62 M, median age 72 (IQR 63–79) years). Examination was complete to the caecum in 96.5% of cases. Capsule endoscopy identified pathology, blood (in the upper gastrointestinal tract [5.6%], small bowel [68.4%], colon [24.1%] or throughout [1.9%]) or both in 54 (47.8%) examinations, guiding further investigation in 30 (55.6%) of these cases. Of the total number of examinations, capsule endoscopy alone or following guided investigation led to new diagnoses in 21 (18.6%, mostly in the small bowel) and therapeutic intervention in 18 (15.9%).

**Conclusions:**

CapsoCam capsule endoscopy had a high completion rate, making new diagnoses in 18.6% of patients and altering management in the majority of this subgroup.

## 1. Introduction

Gastroscopy is recommended within 24 hours of a patient presenting with suspected upper gastrointestinal bleeding but fails to make a diagnosis in 3%–23%, [1–3] raising the possibility that the source of bleeding may be from the small or large bowel. Although melaena is generally considered to occur due to gastrointestinal bleeding proximal to the ligament of Treitz, it may be due to small bowel or ascending colon bleeding if transit is slow. Furthermore, it is not always possible to distinguish melaena from haematochezia. Only 5% of bleeding episodes are thought to arise from the small bowel such that colonoscopy is conventionally performed following a nondiagnostic gastroscopy [2].

Capsule endoscopy examines the small bowel, for which it is a first line investigation. However, although not originally designed to visualise the stomach or the unprepared colon, both are usually seen during transit and so it readily detects the presence of blood in both proximal and distal gastrointestinal tract [3–6]. In patients whose upper and lower flexible endoscopies are nondiagnostic, capsule endoscopy provides a high diagnostic yield in those with active bleeding. It is also much higher if performed within 14 days in those with a self‐limited bleeding episode than if delayed beyond this time frame [6, 7].

Most commercial capsule endoscopy systems require entry of patient details onto a data recorder via a computer. The recorder is mounted in a belt (containing sensor leads) for the patient to wear, and the capsule is activated and paired with the recorder before it is swallowed. Data are then transmitted from capsule to recorder which, on completion of the procedure, is reattached to the computer to convert the raw data to a video format [2–6]. These processes require colocalisation of computer, equipment and patient and training to use them. The CapsoCam Plus (CapsoVision Inc., Saratoga, United States) provides a 360° radial view of the small bowel mucosa using four cameras, each of which have a 90° field of view. Detachment from a magnet on removal from the packaging activates the device which stores imaging data on a flash memory system inside the capsule. This means that it has to be retrieved following excretion in order for the data to be transferred via Bluetooth and converted to a video format. However, there are two advantages of this device: Use requires little staff training, patient preparation or assembly of equipment (there is no data recorder, belt or sensors) so that it can be administered quickly once bleeding occurs. Secondly, a long battery life of 15 hours minimum mitigates against the risk of an incomplete examination in acutely unwell, unprepared patients with impaired motility [8].

## 2. Aims

This study is aimed at evaluating the complete examination rate, diagnostic yield and impact on management of the CapsoCam Plus in patients with acute gastrointestinal bleeding following negative gastroscopy.

## 3. Methods

### 3.1. Design, Setting and Participants

This was an observational study of patients admitted between February 2016 and January 2024 with suspected upper gastrointestinal bleeding to Sheffield Teaching Hospitals NHS Foundation Trust, a district general hospital delivering secondary care to a predominantly urban population of 0.5 million. Patients included were those with overt, suspected upper gastrointestinal tract bleeding in whom gastroscopy had failed to reveal an obvious cause and who subsequently had capsule endoscopy. In the clinical scenario in which there was difficulty distinguishing proximal (melaena) from distal gastrointestinal bleeding (haematochezia), the decision to investigate the mid or distal gastrointestinal tract first was that of the responsible clinician: Some patients whose bleeding was felt to be colonic in origin will not have been referred for capsule endoscopy. All patients were under the care of gastroenterologists, and all endoscopies were performed by consultant gastroenterologists or trainees under their direct supervision using topical anaesthesia with or without light sedation (according to patient choice).

Those in whom examination was normal, or with minor pathologies not thought to be sufficient to explain the presentation had capsule endoscopy using the CapsoCam Plus during the same admission and within 14 days as recommended by European Society of Gastrointestinal Endoscopy (ESGE) guidelines [9]. The decision to refer for capsule endoscopy was entirely at the discretion of the responsible clinician. The protocol required a minimum of 6 hours preprocedural fast (water was allowed). Safety was prioritised over the need for a clean small bowel as it was felt that identifying and localising blood (for subsequent targeted flexible endoscopy) was at least as important as identifying the lesion itself. Therefore, decisions regarding the use of purgatives were left to the referring clinician: Some stable patients were given preprocedure purgatives, and other less stable patients who might have been at risk of further volume depletion were not. The capsule was retrieved and processed, as previously described [8].

All capsule endoscopy videos were reported by a single expert gastroenterologist with experience of reading over 2000 capsule endoscopy videos and who had previously participated in international nomenclature consensus study groups [10]. Anatomical landmarks (first gastric, small bowel and colonic images) were annotated (images are automatically saved with the time after capsule activation), as were all suspected pathological lesions. Pathology was considered to be relevant if thought to be the likely cause of bleeding (analogous to a Saurin classification P2 lesion) [9]. Data regarding benign lesions thought to have little or no bleeding potential (P1 or P0 lesions) are not reported. A written report containing representative images, described according to agreed standards [11] and with a differential diagnosis was provided for the referring clinician who determined subsequent management.

## 4. Data Collection, Variables and Database

### 4.1. Quantitative Data

Data collected included the number of capsule endoscopies performed for overt, suspected upper gastrointestinal bleeding and number of patients in whom they were performed. The time (in days) between onset of bleeding and capsule endoscopy was documented, as well as the performance and timing of any gastroscopy and/or colonoscopy performed prior to capsule endoscopy. Completeness of examination to the caecum and the total gastrointestinal capsule endoscopy imaging time (from oesophageal entry to excretion) were recorded and episodes of capsule retention were sought.

### 4.2. Qualitative Data

Data were also collected retrospectively between February 2023 and November 2024 using online local hospital health care patient information systems and Capsovision capsule endoscopy software. These included patient demographics, nature of bleeding and findings of all forms of endoscopy (gastroscopy, capsule endoscopy, enteroscopy and colonoscopy), relevant radiological investigations and relevant treatments administered (pharmacological, endoscopic and surgical). Outpatient follow‐up was requested for 6–8 weeks posthospital discharge, and final diagnoses recorded were those made by the responsible clinical team in order to cater for further information provided by any postdischarge investigations and/or treatment, rather than using the ICD‐10 diagnostic code assigned by clinical coders on hospital discharge. Diagnostic yield was described as the number (and percentage) of patients with suspected bleeding lesions identified by capsule endoscopy alone and additionally in combination with further investigations performed on the basis of capsule endoscopy findings.

## 5. Statistical Analysis

A database was established using Microsoft Excel for Macintosh Version 16.72, which was used to create graphs and charts. Descriptive statistics (median and IQR) were calculated using SPSS Statistics for Macintosh, Version 28.0 (IBM Corp, Armonk, New York, United States).

## 6. Ethics

Ethics approval was obtained from University of Sheffield (Registration Number: 051067).

## 7. Results

Data were collected for all 113 procedures performed in 106 consecutive patients aged (median [IQR]) 72 (63–79) years (58% male) who had 110 episodes of gastrointestinal bleeding between February 2016 and January 2024. Patients presented with melaena (70.1%), haematochezia (19.1%), both haematemesis and melaena (5.5%), stomal bleeding (3.6%) or haematemesis alone (1.8%). Gastroscopy preceded capsule endoscopy in 91.6% (the remainder having had one or more negative gastroscopies performed for previous similar episodes) and colonoscopy in 16.9%. The median time between onset of bleeding and capsule endoscopy was 9 (5–14) days, and in 109 (96.4%) procedures, gastroscopy was performed 6 (4–13) days before capsule endoscopy.

### 7.1. Capsule Endoscopy Performance and Findings

Capsule endoscopy was complete to the caecum in 109 (96.5%) cases with no device retentions. In the four cases (3.5%) of incomplete capsule endoscopy, the capsule was either not retrieved or data were lost for an unknown reason. Median recording time was 19 hours and 58 minutes (range: 6 hours to 29 hours and 59 minutes). It identified relevant pathology alone, blood alone or both in a total of 54 (47.8%) examinations (Table 1). Relevant pathology considered to be the cause of bleeding was seen in 20 examinations (17.7%), in which blood was also identified in 13, seven patients having clean gastrointestinal tracts. Therefore, most patients (47 [44%]) had evidence of intraluminal blood which was fresh in 30 (64%), dark or altered in nine (19%) and both in eight (17%). Blood (defined as originating from the most proximal section) was localised to the upper gastrointestinal tract in 5.6%, small bowel in 68.4%, colon in 24.1% and throughout in 1.9%.

**Table 1:** Capsule endoscopy findings, subsequent investigations, final diagnoses and management interventions.

Capsule endoscopy category	Subsequent investigation	Final diagnosis/findings	Management impact/intervention
Blood alone (*n* = 34, 30.0%)	Colonoscopy (*n* = 8)	Diverticulosis (*n* = 1)	None
		Angioectasia (*n* = 1)	Clip application
		Haemorrhoids (*n* = 1)	None
	Double balloon enteroscopy (*n* = 5)	Angioectasia (*n* = 2)	Argon plasma coagulation (*n* = 2)
		Neuroendocrine tumour (*n* = 1)	Surgical resection
	Push enteroscopy (*n* = 2)	Dieulafoy lesion (*n* = 2)	Clip application (*n* = 1); adrenaline injection (*n* = 1)
	Gastroscopy (*n* = 3)	Angioectasia (*n* = 1)	Argon plasma coagulation
		Duodenal varix (*n* = 1)	Glue injection
	No further investigation (*n* = 16)∗	No definitive source identified	None
Blood and relevant pathology (*n* = 13, 11.5%)	Colonoscopy (*n* = 1)	Diverticulosis (*n* = 1)	None
	CT small bowel (*n* = 2)	Haemangioma (*n* = 1)	Multidisciplinary team decision to stop anticoagulation
		Dieulafoy lesion (*n* = 1)	Gastroduodenal artery embolisation
	Meckel′s scan + CT small bowel (*n* = 1)	Ulceration at prior Meckel′s surgery anastomosis (*n* = 1)	None
	Gastroscopy (*n* = 2)	Gastric antral vascular ectasia∗ (*n* = 1)	None
		Dieulafoy lesion (*n* = 1)	Clip application and embolisation
	Double balloon enteroscopy (*n* = 5)	Angioectasia (*n* = 4)	Argon plasma coagulation (*n* = 4)
	No further investigation (*n* = 2)	Angioectasia (*n* = 1)	Surgical resection
		Portal hypertensive gastropathy∗ (*n* = 1)	None
Relevant pathology only (*n* = 7, 6.2%)	No further investigation (*n* = 6)	Gastrointestinal stromal tumour∗ (*n* = 1)	None
		Gastric ulcer (*n* = 1)	Proton pump inhibitor therapy
		Haemangioma (*n* = 1)	None
		Duodenal ulcers (*n* = 2)∗	None
		Postvariceal banding ulceration∗ (*n* = 1)	None
	Push enteroscopy (*n* = 1)	No identifiable cause (capsule previously showed angioectasia)	None

*Note:* Asterisk “∗” denotes diagnoses made and treatment instituted on the basis gastroscopy: Capsule endoscopy was performed to exclude other bleeding lesions.

### 7.2. Impact of Capsule Endoscopy Findings on Further Investigation

The localisation of pathology or blood on capsule endoscopy prompted further investigation in 30 (55.6%) of the 54 patients (Table 1). This included repeat gastroscopy (*n* = 5), push enteroscopy (*n* = 3), double balloon enteroscopy (*n* = 10), colonoscopy (*n* = 9), CT imaging only (*n* = 2) and a Meckel′s radioisotope scan (*n* = 1: This patient also had a CT scan).

### 7.3. Diagnoses Made Following Capsule Endoscopy Alone and Following Further Investigation

In total, 21 (18.6% of the total cohort) new diagnoses were made following capsule endoscopy. These included two patients who received treatment based on capsule endoscopy findings alone. In a further 19 patients (16.8% of the total cohort), new diagnoses were made based on further investigation following abnormal capsule endoscopy findings. These included pathologies in the small bowel (duodenal varix [*n* = 1], Dieulafoy lesions [*n* = 4, Figure 1], angioectasia [*n* = 7], ulceration at the site of a previous Meckel′s diverticulum resection [*n* = 1, Figure 2], haemangioma [*n* = 1, Figure 3], neuroendocrine tumour [*n* = 1]) and colon (angioectasia [*n* = 1], haemorrhoids [*n* = 1] and diverticulosis [*n* = 2]).

**Figure 1:**
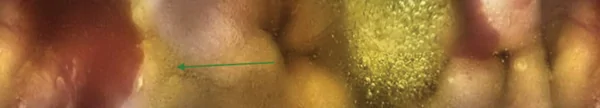
Capsule endoscopy showed active bleeding following which gastroscopy was performed to clip a Dieulafoy lesion in the duodenum.

**Figure 2:**
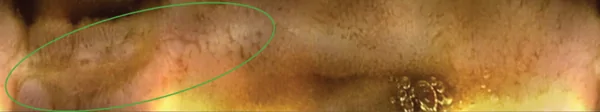
Ulcer at the site of a Meckel′s diverticulum resection.

**Figure 3:**
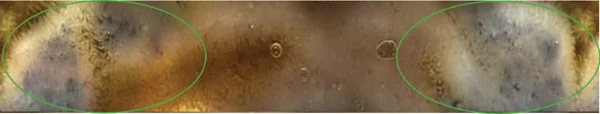
Haemangioma.

A clinical decision was made only to investigate further in the event of rebleeding in the remaining 24 patients.

In six cases, clinicians requested capsule endoscopy because of uncertainty that the pathology identified at prior gastroscopy was the cause of bleeding (duodenal ulceration [*n* = 2, Figure 4], portal hypertensive gastropathy, gastric antral vascular ectasia, gastrointestinal stromal tumour and postvariceal banding oesophageal ulceration [*n* = 1 each]). In these scenarios, capsule endoscopy was used to exclude other causes for the bleeding. Among the six cases, capsule endoscopy confirmed five causes for bleeding. In the case of gastric antral vascular ectasia, the capsule endoscopy could only identify bleeding in the small bowel. This was not included as a new diagnosis made by capsule endoscopy nor an impact on management.

**Figure 4:**
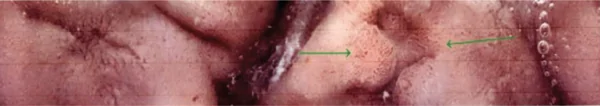
Duodenal ulcers seen on capsule endoscopy, previously missed by gastroscopy.

### 7.4. Impact of Capsule Endoscopy on Patient Management

Capsule endoscopy had a definitive impact on management in 18 patients (33.3% of those with an abnormal study and 16.9% of the total cohort, Table 1). This was based on capsule endoscopy imaging alone in two patients who had proton pump inhibitor therapy for a gastric ulcer (not previously identified by gastroscopy) and surgical resection for angioectasia and in 16 following further investigation: argon plasma coagulation (*n* = 7), surgical resection (*n* = 2), endoclip deployment (*n* = 2), embolization (*n* = 1), both endoclip and embolization (*n* = 1), injection of adrenaline (*n* = 1) or glue (*n* = 1), commencement of a proton pump inhibitor (*n* = 1) and cessation of anticoagulation (*n* = 1).

In one case, capsule endoscopy showed small duodenal ulcers that were not identified on prior gastroscopy (Figure 4). It was decided that these ulcers were thought not to be the cause of the haemorrhage; therefore, they are not included in Table 1.

### 7.5. Outcomes of Patients in Whom Capsule Endoscopy Was Normal

In 59 examinations (52.2%), there were no relevant findings (blood or pathology) on capsule endoscopy following negative gastroscopy [12]. Further investigations were performed in 34 of these patients: pelvic ultrasound (*n* = 1), CT angiography (*n* = 13), abdominal ultrasound (*n* = 4), CT scan (*n* = 13) and nuclear medicine scans (*n* = 2), the results of which can be found in Table 2. The only significant impact on management that further investigation had was splenic artery embolization for treatment of gastric varices not previously identified by gastroscopy: Some patients had their anticoagulation suspended, one with metastatic anal cancer was managed conservatively as were the remainder of the patients with findings of no, or uncertain, significance. Among the 59 cases, 10 had rebleeding (16.9%) within 2 years of discharge from their initial bleed, seven of which cases have been recorded as a procedure in the results section (hence 113 procedures on 106 patients) without further diagnoses being made.

**Table 2:** Further investigation of patients with negative bidirectional endoscopies and negative capsule endoscopy.

Investigation	Findings	Outcome/management
CT angiogram (*n* = 13)	No obvious causative pathology (*n* = 7)	None (*n* = 6)
		Anticoagulant withheld (*n* = 1)
	Haemorrhagic content in the small bowel (*n* = 1)	None (*n* = 1)
	Bleeding vessel in the small bowel (*n* = 3)	None (*n* = 3)
	Bleeding gastric varices (*n* = 1)	Splenic artery embolisation (*n* = 1)
	Large hiatus hernia (*n* = 1)	None (*n* = 1)
Ultrasound abdomen (*n* = 4)	No obvious causative pathology (*n* = 4)	None (*n* = 2)
		Anticoagulant withheld (*n* = 2)
CT abdomen (*n* = 13)	No obvious causative pathology (*n* = 10)	None (*n* = 7)
		Anticoagulant withheld (*n* = 1)
	Diverticulosis (*n* = 3)	None (*n* = 2)
		Anticoagulant withheld (*n* = 1)
Nuclear medicine scan (*n* = 2)	No obvious causative pathology (*n* = 1)	None (*n* = 2)
	Metastatic anal cancer (*n* = 1)	
Ultrasound pelvis (*n* = 2)	No obvious causative pathology (*n* = 1)	Anticoagulant withheld (*n* = 1)
	Uterine soft tissue mass (*n* = 1)	None (*n* = 1)

## 8. Discussion

Capsule endoscopy using the CapsoCam Plus was complete (to the caecum in transit) in 96.5% of examinations and localised the bleeding source in half the patients admitted with suspected gastrointestinal bleeding in whom gastroscopy did not provide a clear diagnosis. In those with an abnormal study (showing blood with or without a causative lesion), it guided further investigation in 26.5% leading to new diagnoses in 18.6% (made either at capsule endoscopy or following further investigation) of the total cohort and a change in management in 16.9%.

A recent meta‐analysis found that complete capsule endoscopy to the caecum occurred in 90.6% of patients with suspected small bowel bleeding [10]. However, illness impairs gastrointestinal motility and ability to comply with bowel preparation regimens: Studies on hospitalised [11] and those with overt gastrointestinal bleeding [12] reported complete small bowel examination rates of only 77.1% and 81.9%, respectively. This is of importance as the time the capsule spends imaging the small bowel correlates with diagnostic yield [13–15]. The CapsoCam imaging time of almost 20 hours could potentially explain the 96.5% completion rate in this study making it particularly suitable in this clinical scenario.

Previous studies suggest that the small bowel is the source of bleeding in approximately 5% of patients [2] implying the need for colonoscopy in patients whose gastroscopy is nondiagnostic. In fact, we previously found that small bowel lesions were the source of bleeding in 18% of patients referred with suspected upper gastrointestinal bleeding, and of the 47 patients in whom capsule endoscopy identified blood in this study, it was localised to small bowel in 68.4% [4]. In a similar vein, Schlag et al. [3] identified positive findings (blood or a lesion) in 15 of 20 patients with bleeding following negative gastroscopy, 73% of whom had a small bowel cause. We found that in only four examinations were lesions seen within the colon thought to be causing the bleeding and blood was seen in the colon in just 24.1% of examinations. These data are consistent with the findings in this study suggesting that the small bowel is significantly more likely to be the source than the colon. Capsule endoscopy would be a more appropriate test than colonoscopy in patients with suspected upper gastrointestinal bleeding if gastroscopy is nondiagnostic.

Studies report a very variable diagnostic yield of second look gastroscopy in patients with gastrointestinal bleeding (between 2% and 75%). The ESGE does not recommend this practice before capsule endoscopy, which does not appear to be cost‐effective, although the American guidelines favour doing so unless capsule endoscopy is more expedient [2, 11, 16, 17]. Our study shows that lesions in the small bowel are far more common than proximal lesions and delaying capsule endoscopy to repeat gastroscopy would risk reducing diagnostic yield [6, 17]. An appropriate approach might be to consider repeat gastroscopy in patients with haematemesis (which is less likely to originate from distal to the duodenum than melaena) or in whom first inspection was suboptimal due to the presence of luminal content (food or blood), poor tolerance or operator inexperience: Capsule endoscopy should be offered to the remaining patients at the earliest convenience [2, 11].

Recent literature goes further, positioning capsule endoscopy as the first line test to determine if flexible endoscopy is needed, and if so, whether a proximal or distal approach is appropriate. Capsule endoscopy as a first investigation in all patients with suspected GI bleeding during the COVID‐19 pandemic had a better diagnostic yield with fewer invasive procedures compared with a postpandemic cohort undergoing standard investigation [18]. The same outcomes were described in a prospective, single‐centre study when comparing combined small and large bowel capsule endoscopy with colonoscopy in patients with suspected GI bleeding in whom gastroscopy was noncontributory [19]. Diagnostic yield was also greater in those randomised to capsule endoscopy as the first investigation of nonhaematemesis GI bleeding compared with standard care [20]. These authors found no difference in cost of the two care pathways, but projected that while length of stay could have been reduced in both arms, it was potentially greater in those receiving capsule endoscopy compared with standard care [21]. Therefore, in patients with nondiagnostic gastroscopy, capsule endoscopy should be considered. Further research is needed to clarify the role of capsule endoscopy in the different clinical scenarios; however, as a first line test for all patients with suspected acute GI bleeding, either prior to or following negative gastroscopy or just those with nonhaematemesis bleeding. Multicentre studies would be needed to confirm preliminary findings of the single‐centre studies and further cost analyses of the different investigative pathways.

A key limitation to consider with this study is the large time delay between upper gastrointestinal bleed and initial capsule endoscopy. The 9‐day median delay in this study was not in keeping with the current ESGE recommendation of 48 hours [11], though it does acknowledge that this is not always feasible so the capsule should be done as soon as possible.

Another limitation of the study was that referral for capsule endoscopy was at the behest of the responsible clinician, making the study prone to selection bias so our results may not be a comprehensive review of the experience of all patients with suspected upper gastrointestinal bleeding whose gastroscopy was noncontributory. How this might have affected diagnostic yield and outcomes is uncertain. On the one hand, the ESGE recommends capsule endoscopy as soon as possible (and ideally within 48 hours) after the bleeding episode to maximise diagnostic yield [22]. On the other, a median delay of 9 days in our cohort might have selected out those with minor bleeding due to benign and rapidly healing lesions, the identification of which may not impact management. Such low‐risk patients are not always referred for further investigation in routine practice, and the pragmatic approach in this study of leaving management decisions to the responsible clinicians means that the results are likely to be generalisable to experience in other acute hospital centres. The ESGE acknowledges that organisational factors might render capsule endoscopy within a 48‐hour timeframe unfeasible [22]; therefore, the simplified set‐up and limited training requirements of the CapsoCam Plus may reduce some logistical barriers. Other capsule endoscopy systems also offer practical advantages that may facilitate timely deployment, though differences between these variations were not formally assessed in this study.

A further possible limitation is that the CapsoCam Plus stores data in an on‐board flash memory system and has to be retrieved following excretion. The possibility that some studies were lost due to nonretrieval of the device cannot be excluded. Bowel preparation, specifically the use of purgatives, was guided by the referring clinician, depended on the clinical condition of the patient and was therefore not consistent.

Finally, capsule endoscopy diagnoses were those of a single expert capsule endoscopy reader. Although this individual had previously participated in studies of interobserver variability in lesion description, no such exercises were performed in this study [10]. This therefore reduces the generalisability of our findings because clinical practice uses multiple heterogeneous readers of differing experience, who may interpret findings differently to the single reader used here. As a result, diagnoses, further investigations and decisions for subsequent treatments may differ.

## 9. Conclusions

Capsule endoscopy using the CapsoCam in patients with overt suspected upper gastrointestinal bleeding had a very high complete examination rate, which is likely to be related to a 20‐hour recording time. It achieved a high diagnostic yield, most pathology occurring in the small bowel and the identification of which guided management in most patients, supporting the recommendation that capsule endoscopy should be performed after a nondiagnostic gastroscopy. Patients presenting with suspected upper gastrointestinal bleeding in whom gastroscopy is noncontributory should be referred for subsequent capsule endoscopy to localise bleeding and/or a potential causative lesion within the small bowel. Ease of delivery and the long battery life of the Capsocam Plus lends itself well to the emergency scenario.

## Author Contributions

Reena Sidhu and Mark E. McAlindon devised the study. Calvin M. Johnson, Veena Shivakumar and Stanley W. McKenzie collated and analysed the data. Calvin M. Johnson and Mark E. McAlindon wrote the main manuscript text and prepared Figures 1, 2, 3 and 4 and Table 1.

## Funding

No funding was received for this manuscript.

## Disclosure

All authors reviewed the manuscript.

## Conflicts of Interest

The authors declare no conflicts of interest.

## Supporting information


**Supporting Information** Additional supporting information can be found online in the Supporting Information section. File S1: STROBE statement—checklist of items that should be included in reports of cohort studies.

## Data Availability

The datasets generated and/or analysed during the current study are available from the corresponding author on reasonable request.
